# Tilmanocept as a novel tracer for lymphatic mapping and sentinel lymph node biopsy in melanoma and oral cancer

**DOI:** 10.1111/ans.17868

**Published:** 2022-07-18

**Authors:** Derek Mwagiru, Pranav Shivashankar, Eva Wong, David Farlow, Brad Cambden, Muzib Abdul‐Razak

**Affiliations:** ^1^ Department of Surgical Oncology and Head and Neck Surgery Crown Princess Mary Cancer Centre Sydney New South Wales Australia; ^2^ Department of Nuclear Medicine Westmead Hospital Sydney New South Wales Australia; ^3^ Faculty of Medicine, Department of Surgical Oncology and Head and Neck Surgery Crown Princess Mary Cancer Centre, University of Sydney Sydney New South Wales Australia

**Keywords:** lymphoscintigram, melanoma, oral cancer, radioactive tracer, sentinel lymph node biopsy

## Abstract

**Background:**

Sentinel lymph node biopsy (SLNB) has been pivotal for pathological assessment of nodal status in cutaneous melanoma (CM) and oral cavity squamous cell carcinoma (OCSCC) thus crucial for staging. An ideal agent for lymphatic mapping should have a standardized preparation, appropriate accumulation in first‐echelon nodes and no side effects. Tilmanocept, a CD206‐receptor targeted novel radiotracer fulfils these properties. This study investigated Tilmanocept for lymphoscintigraphy and intraoperative identification of sentinel lymph nodes (SLN) in CM and OCSCC.

**Methods:**

This prospective cross sectional study examined patients who presented to Crown Princess Mary Cancer Centre, Westmead Hospital, Sydney. Patients had biopsy proven tumours with clinically and radiologically negative regional lymph nodes. Tilmanocept guided lymphoscintigraphy was followed by intraoperative SLNs identification via handheld gamma probe. Primary endpoints were detection and retrieval rate of SLNs while secondary endpoints included pathological status of SLNs.

**Results:**

Thirty‐five patients were included (26 with CM and 9 with OCSCC) with the most common primary tumour site for CM on the extremities (33.3%). Lymphoscintigraphy with Tilmanocept identified at least 1 SLN (sensitivity 100%) in all patients. SLNs were retrieved in all of patients intraoperatively (100% retrieval rate) with positive nodes found in 20% of patients. Tilmanocept also demonstrated 100% tissue specificity, with lymph nodal tissue confirmed histologically, with no false positives.

**Conclusion:**

Tilmanocept is a reliable radiotracer for assessing the nodal status in patients with CM and OCSCC. Our group is the first to evaluate the use of Tilmanocept in the Australian setting, adding to the limited studies worldwide.

## Graphical abstract

Our study is evaluation of novel radioactive tracer Tilmanocept in performing sentinel node biopsy in cutaneous melanoma and oral cancer. Tilmanocept identified at least one sentinel node in all patients and this was retrieved leading to a 100% sensitivity in the technique.Tilmanocept is a reliable radio tracer for assessing the nodal status in these patients.

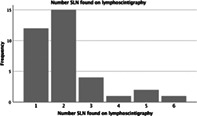

## Introduction

Cutaneous melanoma (CM) is a growing global health problem with an increasing incidence worldwide^1^ and presents a staging challenge, as 90% of cases have no clinical evidence of metastatic disease in the regional lymph nodes.^2^ In early OCSCC in spite of no clinical or radiological evidence of cervical nodal metastases^3^, ^4^ occult metastases occurs in 23–43%^4^ of patients. The assessment of regional lymph nodes is crucial as presence of nodal metastases is a strong prognostic factor for recurrence and survival.^2^

Sentinel lymph node biopsy (SLNB) has been routinely used as a minimally invasive technique in staging and management of CM and in OSCC in selected centres. SLNB if positive identifies patients who may benefit from systemic therapeutic interventions.^5^ SLNB for OCSCC in large multicentre trials such as Schilling *et al*. have reported sensitivity, negative predictive value (NPV), and a false negative rate of 86%, 95%, and 14%, respectively.^4^ Recently three randomized trials have been published which show SLNB to be as reliable as neck dissection while significantly reducing the morbidity in the management of early OCSCC.^4^, ^6^, ^7^

Current methods of SLNB use administration of radioactive tracers followed by lymphoscintigraphy to identify the sentinel lymph node (SLN) along with single photon emission computerized tomography (SPECT) to help with anatomic localisation of the node. The most commonly used radiotracer is ^99m^Tc‐Labelled sulphur colloid, which has a large molecular weight thus sequestrating in the SLN. Due to the large molecular size of this tracer there is delayed clearance from the primary site thus masking SLNs which are very close to the injection site on the lymphoscintigram. Operative identification of the SLNs also becomes difficult when the relevant node is close to the primary injection site due to the ‘shine through’ phenomenon. Several studies examining the use of SLNB in OCSCC have shown false negative rates reaching nearly 15% in multicentre trials especially when the SLN is close to the primary as it occurs in floor of mouth cancers.^8^ Agrawal *et al*. hypothesised that this is partly due to the radiolabelled colloids lacking specific binding sites to the nodes.^8^ Tilmanocept is a novel radiopharmaceutical designed to overcome these limitations of conventionally used radiotracers.^9^

Properties of an ideal radiotracer for lymphatic mapping include a standardized preparation, good safety profile, rapid uptake and optimal accumulation in first‐echelon nodes, rapid clearance from the injection site and minimal pass through to second echelon (non‐sentinel) lymph nodes.^5^, ^9^ [^99m^ Tc] Tilmanocept is a novel synthetic radiopharmaceutical, consisting of molecules of mannose and diethylene triamine penta‐acetic acid (DTPA), that accumulate in lymphatic tissue by selectively targeting and binding to CD 206 mannose receptors on the surface of resident macrophages and dendritic cells, which are found in high concentrations in lymph nodes.^9^ Given its low molecular weight, it is rapidly is taken up by first echelon lymph nodes and the molecular binding limits migration to second echelon nodes.^9^ Tilmanocept additionally requires no manipulation after the injection^5^ and is associated with decreased injection site pain.^10^

The aim of this study was to investigate Tilmanocept in a prospective study to delineate its application, suitability and reliability in CM and OCSCC in an Australian setting.

## Methods

Patients presenting to Crown Princess Mary Cancer Centre, Westmead hospital, Sydney, with CM or OCSCC were recruited prospectively. Participants included in the study were patient's ≥18 years with biopsy proven primary CM and OCSCC who were clinically and radiologically node negative and candidates for SLNB as a part of their management. Patients who refused staging, patients who had a life expectancy of less than 5–10 years, patients with radiological or palpable regional nodes or presence of distant metastases were excluded. As the Australian Therapeutic goods association did not approve Tilmanocept at the time of the study, approval for each participant was through the New Interventional Procedures Assessment Committee (NIPAC) and individual Special Access Scheme (SAS). Following this HREC Westmead approved the study (QA approval number 2005‐07).

### Procedure

All patients underwent preoperative lymphoscintigram with 250 micrograms of Tilmanocept (Lymphoseek—Cardinal Health) radiolabelled with approximately 100 MBq technetium 99mTc administered as 4 quadrant injections around the tumour (intradermal for melanoma and submucosal for OCSCC patients respectively). Patients scheduled for the same day of surgery received Tilmanocept with approximately 20 MBq of technetium 99mTc while patients who had their operation scheduled for the day after had 60–80 MBq Technetium 99mTc to allow for appropriate decay. Following administration, patients underwent lymphoscintigraphy with dynamic imaging followed by sequential planar imaging then SPECT‐CT of the relevant nodal basin(s). Skin markings were placed on the identified nodes with patient in operative position based on the SPECT scan done at the same time. In the operating theatre blue dye (Patent Blue V—Guerbet) was injected peri‐tumour for melanoma patients to aid the location of the lymph node and gamma probe (Neoprobe—Devicor Medical Products) was used to identify and remove the radioactive sentinel lymph nodes. Radioactivity counts were recorded for all identified nodes, which were then sent for histological examination. SLNs were identified by the 3σ rule and this corresponds to a 99.7% confidence limits. This rule states that the SLN must exceed the background count plus three times the standard deviation of the background count.^1^ The background activity of the area was confirmed as <10% of the most radioactive node at the end of the operation to confirm complete removal of all SLNs. SLNs were processed using standard SLNB protocol at our institution. Briefly, SLNs <2 mm were analysed as a whole; SLNs 2–5 mm were cut through the hilum, and nodes >5 mm were cut into 3 mm slices and processed. Serial sectioning of each slice was then performed at every 200 μm. Light microscopic examination of tissue sections was carried out after routine haematoxylin/eosin (HE) stain and immunohistochemistry was done with pan‐cytokeratin antibodies (AE1/AE3) for OCSCC and S‐100 and HMB‐45 for CM.

### Data collection

Data collection included patient demographics, lymphoscintigram data, operative findings, pathology of the sentinel nodes and clinical follow up. For CM the subtype, Breslow thickness, mitotic rate, presence of ulceration and Clarke level were also recorded. For OCSCC primary tumour characteristics were included. Histopathological data including total nodal involvement, size of largest nodal metastases and extracapsular spread were analysed. The primary outcomes were SLN identification rate on the lymphoscintigram and SLN retrieval rate during surgery. Secondary outcomes included pathological status of SLNB (recorded as either positive or negative) and patient safety after injection, during surgery and 24 h and 1‐week post‐surgery.

### Statistical analysis

IBM SPSS Statistics version 26 was used to analyse the data. Categorical variables were summarized using frequencies and percentages. The mean, standard deviation (SD), minimum, maximum and quartiles were used to summarize continuous variables. Chi‐squared or exact permutation tests were used to test for association between categorical variables. The percentage of patients in whom the same number of SNL were found in surgery and on lymphoscintigraphy was calculated together with its 95% confidence interval (95% CI). Similarly, for both SLNs found in surgery and for those found on lymphoscintigraphy, the percentage and 95% CI of SLNs which were positive was calculated by Breslow thickness group, mitotic index group, lymphovascular invasion status and ulceration status.

## Results

A total of 35 patients were included in the study who fulfilled the CM and OCSCC selection criteria and needed staging with SLNB. We included 26 CM patients (74.3%) and 9 OCSCC patients (25.7%), all of which were biopsy proven. Patients were mostly males comprising of 65.7% with a mean age of 62.9 years. Table 1 shows patient demographics and CM/OCSCC tumour characteristics and Table 2 the SLN variables.

**Table 1:** Patient demographics and tumour variables

Variable	Values taken	*n*	%
Type	Oral cavity squamous cell carcinoma	9	25.7
	Melanoma	26	74.3
Gender	Male	23	65.7
	Female	12	34.3
Number of comorbidities	≤2	4	16.0
	>2	21	84.0
Smoking	No	8	40.0
	Yes	12	60.0
Alcohol use	No	8	33.3
	Yes	16	66.7
History of melanoma	No	18	78.3
	Yes	5	21.7
Primary site—Melanoma	Extremity	11	31.4
	Head/neck	9	25.7
	Back	6	17.1
Primary site—OCSCC	Tongue	7	20
	Mandible	1	2.9
	Buccal	1	2.9
Melanoma subtype	Desmoplastic	1	2.9
	Lentigo maligna melanoma	3	8.6
	Nodular	10	28.6
	Superficial spreading	8	22.9
	Not otherwise specified	4	11.4
Ulceration	No	16	61.5
	Yes	10	38.5
Breslow thickness (grp)	≤2	15	57.7
	>2 and <4	9	34.6
	≥4	2	7.7
Mitotic index (grp)	≤7	12	57.1
	8–15	7	33.3
	≥16	2	9.5
Lymphovascular invasion	No	25	89.3
	Yes	3	10.7
Perineural invasion	No	24	85.7
	Yes	4	14.3
	Yes	3	8.6
>12 h injection to lymphoscintigraphy	No	18	51.4
	Yes	17	48.6

**Table 2:** Sentinel lymph node characteristics

Variable	*n*	Minimum	Percentile 25	Median	Percentile 75	Maximum	Mean	SD
Age	35	29.0	51.0	65.0	71.0	90.0	62.9	14.0
Number SLN found on lymphoscintigraphy	35	1	1	2	2	6	2	1
Breslow thickness	26	0.7	1.1	1.6	2.5	10.0	2.2	1.9
Mitotic index	21	0.0	1.0	5.0	11.0	21.0	6.7	6.5
SN radiation count	29	2256	6270	19 307	45 870	93 147	28 527	26 489
Background count	23	1	7	27	80	347	56	81
Number SLN found in surgery	35	1	2	3	4	6	3	1
Number SLN positive	35	0	0	0	0	4	0	1

**Fig. 1:**
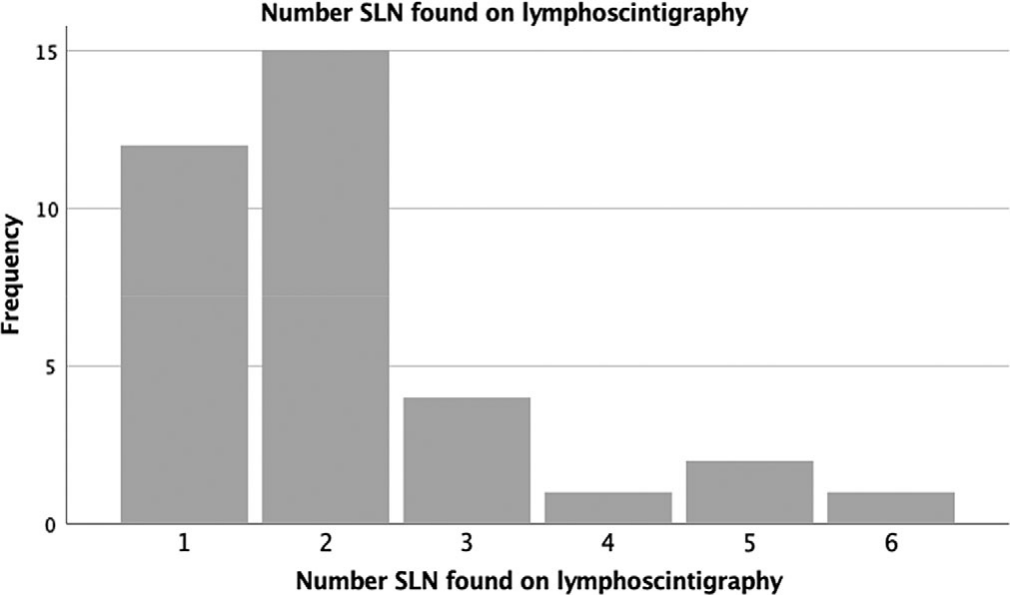
Frequency distribution of sentinel lymph nodes on lymphoscintigraphy.

**Fig. 2:**
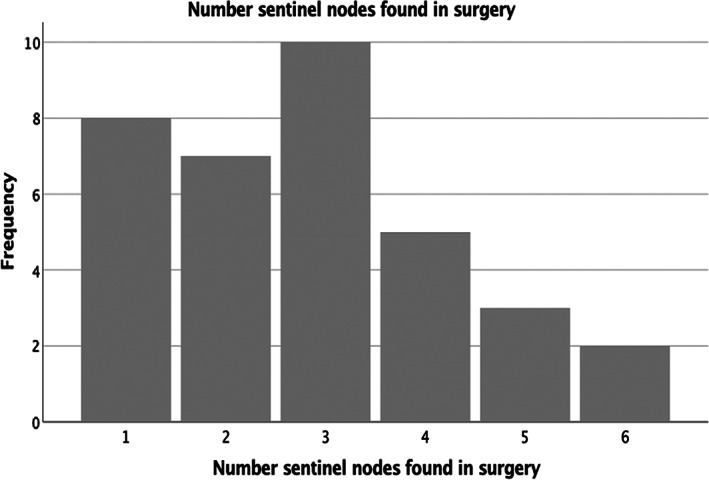
Frequency distribution of sentinel lymph nodes retrieved during surgery.

**Fig. 3:**
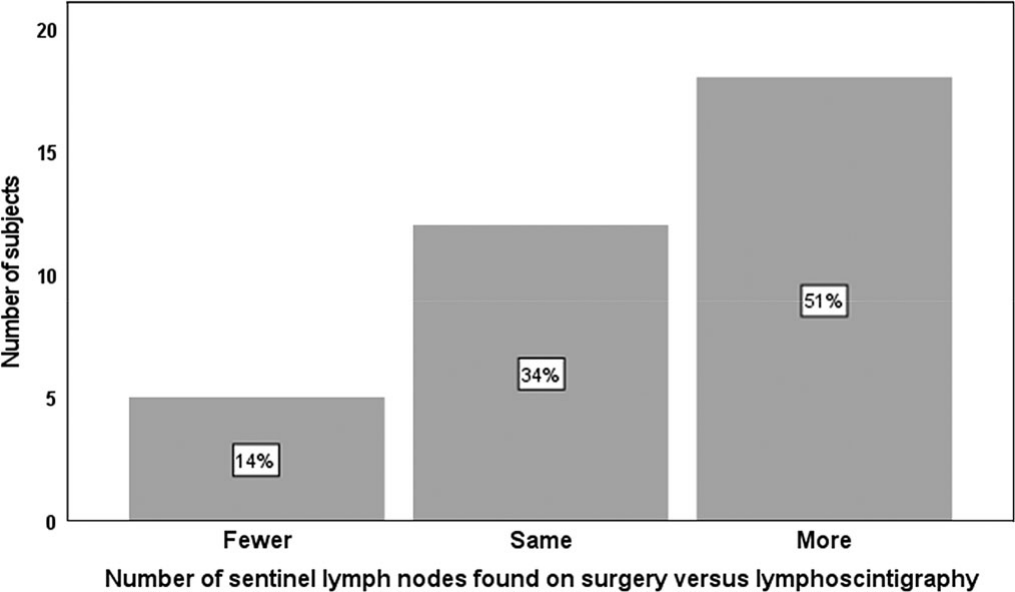
Sentinel lymph nodes found in lymphoscintigraphy versus surgery.

Tilmanocept was administered and the surgery performed on the same day in 51% of patients while 49% of patients had the surgery the next day due to theatre availability. There was no difference in the SLN detection or retrieval rate between these two groups. Tilmanocept guided lymphoscintigraphy was able to identify at least 1 SLN (sensitivity 100%, 95%CI (89.9–100%) in all 35 patients and appropriate lymph node (s) were retrieved in all those patients. Figs. 1 and 2 depict the number of lymph nodes detected by lymphoscintigraphy and the actual number of lymph nodes retrieved during surgery. The higher retrieval number in surgery is due to better spatial resolution of the handheld gamma probe operated by the surgeon as opposed to lymphoscintigram (Fig. 3). There were no adverse events due to Tilmanocept injection peri operatively and the injections were well tolerated by the patients.

SLNs were located mostly in the neck in 45.7%, followed by the axilla in 17.1% and the groin in 22.9%. In most patients, 3 SLNs were retrieved (26.7%), while 23.3% of patients had 1 node and 20% had 2 SLNs retrieved. A total of 99 SLNs were resected in 35 patients out of whom 13 were positive resulting in a SLN positivity rate of 13% (95% CI 7.8% ‐ 21.2%). Table 3 depicts the relationship of SLN positivity to crucial pathological parameters of CM namely Breslow thickness, mitotic index, lymphovascular invasion and ulceration. In terms of tissue origin specificity, 100% of the tissues identified by Tilmanocept radiotracer were confirmed histologically as lymph nodes with no false positives.

**Table 3:** Relationship of SLN positivity to pathological parameters for melanoma patients (*n* = 26)

Variable	Values taken	Number SLNs found in surgery	Number positive SLNs	% Positive SLNs	95%CI
Breslow thickness (mm)	≤2	45	0	0.0	(0–7.9%)
	>2 and <4	24	6	25.0	(12.0–44.9%)
	≥4	6	1	16.7	(3.0–56.4%)
Mitotic index (number of mitoses per 10 hpf)	≤7	38	0	0.0	(0–9.2%)
	8–15	20	5	25.0	(11.2–46.9%)
	≥16	5	2	40.0	(11.2–76.9%)
Lymphovascular invasion	No	74	8	10.8	(5.6–19.9%)
	Yes	10	3	30.0	(10.8–60.3%)
Ulceration	No	43	2	4.7	(1.3–15.5%)
	Yes	28	8	28.6	(15.3–47.1%)
All 35 patients		99	13	13.1	(7.8–21.2%)

## Discussion

SLNB is widely accepted as an important clinical step in staging CM^5^ and is also a validated procedure to stage the neck in OCSCC.^11^ Despite this, different radio‐colloids and Vital blue dyes have resulted in a non‐standardized method of localizing and retrieving SLNs. Tilmanocept was developed to overcome limitations of radio‐colloids and acts by selectively targeting and binding CD206 receptors on the surface of macrophages and dendritic cells.^9^ The use of Tilmanocept as a radiopharmaceutical in CM and OCSCC has not been studied in Australia to date. In this prospective study in patients with CM and OCSCC we have shown that Tilmanocept can accurately identify SLNs on lymphoscintigraphy, aid in intraoperative detection with gamma probe and provide relevant specimens for histopathological examination.

There has been limited research on the use of Tilmanocept in CM or OCSCC, however initial studies have shown promising results. In CM a Phase III trial in 15 centres with 154 patients compared Tilmanocept with Vital blue dye for identification of SLN in clinically node negative patients.^5^ In this trial the primary endpoint was concordance, which was the proportion of blue nodes, detected by Tilmanocept, with 90% concordance being minimal level accepted.^5^ This trial found that Tilmanocept had a 98.7% concordance. Tilmanocept identified the appropriate SLN in more patients, increased SLN yield in any given patient and identified more melanoma‐containing nodes than vital blue dye, with no serious adverse events.^5^ In terms of OCSCC, multicentre validation studies of 85 patients utilizing Tilmanocept for SLNB have shown identification rate of 97.6%, false negative rate of 2.56% with negative predictive values of 97.8%.^8^ This study found that when compared to sulphur colloid, Tilmanocept had a significantly lower false negative rate and higher accuracy of node retrieval.^8^ Agrawal *et al*. showed that sulphur colloids were retained for prolonged periods within the injection site, which contributed to shine through effect.^8^ The shine through effect becomes an issue in head and neck where the nodes are in close proximity to the primary. Even resecting the primary prior to assessing the SLNs does not obviate the shine through effect due to tracer retention at the primary and as a result the false negative rate in this location has been traditionally higher. Toom *et al*. in a small prospective analysis of 20 patients reported that the higher injection site clearance was present in Tilmanocept when compared to sulphur colloids and aids in better detection of these SLNs close to the primary.^11^ Tilmanocept is also proposed to stay in the SLN longer with less migration to second echelon nodes due to molecular adhesion (opposed to molecule size limited trapping as in sulphur colloid) thus increasing the reliability of the technique.

In the current study Tilmanocept based lymphoscintigraphy was able to identify at least one radioactive SLN in 100% of patients, which was retrievable in surgery in all those patients with subsequent confirmation histologically with no false positives. A prospective Phase II clinical trial reviewing 47 CM and 31 breast cancer patients demonstrated the safety and efficacy of Tilmanocept. In this study lymphoscintigraphy identified a hot spot in 94.5% of patients prior to surgery and in 96.2% of patients one regional SLN was identified.^12^ Similarly, Sondak *et al*. studied 154 patients injected with Tilmanocept and Vital blue dye and found 97.4% of patients had a radioactive node identified intraoperatively.^5^ Agrawal *et al*. replicated the reliability of Tilmanocept as a radiotracer in a multicentre trial of 83 patients, where they were able to identify at least 1 SLN in 97.6% of patients intraoperatively.^8^

The ability of Tilmanocept to identify positive lymph nodes is imperative in the prognostication of survival, regional recurrence and overall management. Leong *et al*. found that the overall proportion of Tilmanocept positive nodes containing metastatic disease was 13.7%^12^ in patients with CM and breast cancer. This compares well with our current results which show a 13% SLN positivity in CM and OCSCC. Further studies by Silvestri *et al*., which compared Tilmanocept to sulphur colloid and vital blue dyes validated Tilmanocept ability to identify positive lymph nodes. In this case series, Tilmanocept was compared to sulphur colloid in an age, sex and primary tumour characteristic matched population. This study found that there was no statistical difference between the two groups in identifying positive lymph nodes.^10^ Furthermore, a multicentre Phase III trial in oral squamous cell carcinomas demonstrated high overall accuracy (with negative predictive value of 97.8%) and low false negative rates (2.56%) for Tilmanocept.^8^ Tilmanocept has been compared to a meta‐analysis of six studies with Technetium nanocolloid for breast cancer where Tilmanocept was superior in both endpoints of SLN localisation rate and degree of SLN localisation.^13^ The current study adds to this growing body of evidence that Tilmanocept can accurately identify positive SLNs to aid in staging and management of CM and OCSCC.

Our study, despite demonstrating promising results, has several limitations. The first limitation is the small sample size. The cohort of 35 patients we believe provides at least an indication on the feasibility of Tilmanocept as a radiopharmaceutical in the Australian setting. The current study when compared to previous published research, still provides similar results and conclusions. This study is also limited by the study design. In the current study, Tilmanocept was not directly compared to another radiopharmaceutical whereas previous studies have compared Tilmanocept to Vital blue dyes and sulphur colloid. This pilot study can be improved in further research by comparing Tilmanocept to sulphur colloids in an age, sex and primary tumour characteristic matched population.

In conclusion this study, though small in number, provides evidence that Tilmanocept based lymphoscintigraphy is a feasible and accurate method for assessing the nodal status in patients with CM and OCSCC. All patients had an identifiable lymph node on lymphoscintigraphy, intraoperatively and on histology. Our research is the first to evaluate the use of Tilmanocept in the Australian setting and adds to the growing evidence worldwide.

## Conflict of interest

None declared.

## Author contributions

**Derek Mwagiru:** Data curation; formal analysis; writing – original draft. **David Farlow:** Investigation; supervision; validation. **Pranav Shivashankar:** Data curation; writing – original draft; writing – review and editing. **Eva Wong:** Conceptualization; investigation; methodology. **Bradley Camden:** Investigation; project administration; supervision; validation. **Muzib Abdul‐Razak:** Conceptualization; investigation; methodology; validation; project administration; data curation; writing and editing original draft; formal analysis and supervision.

## References

[1] Tardelli E , Mazzarri S , Rubello D *et al*. Sentinel lymph node biopsy in cutaneous melanoma: standard and new technical procedures and clinical advances. A systematic review of the literature. Clin. Nucl. Med. 2016; 41: e498–507. 27749418 10.1097/RLU.0000000000001370

[2] Morton DL , Wen DR , Wong JH *et al*. Technical details of intraoperative lymphatic mapping for early stage melanoma. Arch. Surg. 1992; 127: 392–9. 1558490 10.1001/archsurg.1992.01420040034005

[3] Frydrych AM , Slack‐Smith LM , Parsons R , Threlfall T . Oral cavity squamous cell carcinoma: characteristics and survival in aboriginal and non‐aboriginal Western australians. Open Dent. J. 2014; 8: 168–74. 25328552 10.2174/1874210601408010168 PMC4200744

[4] Schilling C , Stoeckli SJ , Haerle SK *et al*. Sentinel European node trial (SENT): 3‐year results of sentinel node biopsy in oral cancer. Eur. J. Cancer 2015; 51: 2777–84. 26597442 10.1016/j.ejca.2015.08.023

[5] Sondak VK , King DW , Zager JS *et al*. Combined analysis of phase III trials evaluating [(9)(9)mTc]tilmanocept and vital blue dye for identification of sentinel lymph nodes in clinically node‐negative cutaneous melanoma. Ann. Surg. Oncol. 2013; 20: 680–8. 23054107 10.1245/s10434-012-2612-z PMC3560941

[6] Civantos FJ , Zitsch RP , Schuller DE *et al*. Sentinel lymph node biopsy accurately stages the regional lymph nodes for T1‐T2 oral squamous cell carcinomas: results of a prospective multi‐institutional trial. J. Clin. Oncol. 2010; 28: 1395–400. 20142602 10.1200/JCO.2008.20.8777 PMC2834497

[7] Flach GB , Bloemena E , Klop WM *et al*. Sentinel lymph node biopsy in clinically N0 T1‐T2 staged oral cancer: the Dutch multicenter trial. Oral Oncol. 2014; 50: 1020–4. 25164950 10.1016/j.oraloncology.2014.07.020

[8] Agrawal A , Civantos FJ , Brumund KT *et al*. [(99m)Tc]Tilmanocept accurately detects sentinel lymph nodes and predicts node pathology status in patients with oral squamous cell carcinoma of the head and neck: results of a phase III multi‐institutional trial. Ann. Surg. Oncol. 2015; 22: 3708–15. 25670018 10.1245/s10434-015-4382-x PMC4565859

[9] Surasi DS , O'Malley J , Bhambhvani P . 99mTc‐Tilmanocept: a novel molecular agent for lymphatic mapping and sentinel lymph node localization. J. Nucl. Med. Technol. 2015; 43: 87–91. 25956693 10.2967/jnmt.115.155960

[10] Silvestri C , Christopher A , Intenzo C *et al*. Consecutive case series of melanoma sentinel node biopsy for lymphoseek compared to sulfur colloids. J. Surg. Res. 2019; 233: 149–53. 30502241 10.1016/j.jss.2018.07.042

[11] Den Toom IJ , Mahieu R , Van Rooij R *et al*. Sentinel lymph node detection in oral cancer: a within‐patient comparison between [(99m)Tc]Tc‐tilmanocept and [(99m)Tc]Tc‐nanocolloid. Eur. J. Nucl. Med. Mol. Imaging 2021; 48: 851–8. 32839855 10.1007/s00259-020-04984-8 PMC8036184

[12] Leong SP , Kim J , Ross M *et al*. A phase 2 study of (99m)Tc‐tilmanocept in the detection of sentinel lymph nodes in melanoma and breast cancer. Ann. Surg. Oncol. 2011; 18: 961–9. 21331809 10.1245/s10434-010-1524-z PMC3071527

[13] Christopher AT , Frederick OC , Webdy LM *et al*. The efficacy of Tilmanocept in sentinel lymph node mapping and identification in breast cacncer patients: a comparative review and meta‐analysis of ^99^Tc‐labeled nanocolloid human serum albumin standard of care. Clin. Exp. Metastasis 2012; 29: 681–6. 22729510 10.1007/s10585-012-9497-x

